# Application of Fe_2_O_3_ nanoparticles improves the growth, antioxidant power, flavonoid content, and essential oil yield and composition of *Dracocephalum kotschyi* Boiss.

**DOI:** 10.3389/fpls.2024.1475284

**Published:** 2024-10-09

**Authors:** Parisa Khanizadeh, Hasan Mumivand, Mohamad Reza Morshedloo, Filippo Maggi

**Affiliations:** ^1^ Department of Horticultural Sciences, Faculty of Agriculture, Lorestan University, Khorramabad, Iran; ^2^ Department of Horticultural Science, Faculty of Agriculture, University of Maragheh, Maragheh, Iran; ^3^ Chemistry Interdisciplinary Project (ChIP) Research Center, School of Pharmacy, University of Camerino, Camerino, Italy

**Keywords:** iron, plant pigments, FRAP, citral, phenylalanine ammonia-lyase

## Abstract

*Dracocephalum kotschyi* Boiss., an endemic and endangered medicinal and aromatic plant in Iran, showcases distinct botanical characteristics and therapeutic promise. According to the IUCN grouping criteria, this plant is facing challenges due to overcollection from its natural habitats. To address this issue, there is an increasing inclination towards cultivating this species within agricultural systems. This study aimed to evaluate the impact of applying Fe_2_O_3_ nanoparticles (NPs) at varying concentrations (50, 100, and 200 mg L^-1^), as well as bulk Fe_2_O_3_ at the same concentrations, on the growth, essential oil production, antioxidant capacity, total phenol, and flavonoid content of *D. kotschyi*. The foliar application of 100 and/or 200 mg L^-1^ of Fe_2_O_3_ NPs resulted in the greatest leaf length and dry weight, while Fe_2_O_3_ NPs at the level of 100 mg L^-1^ led to the highest leaf/stem ratio. Additionally, spraying 200 mg L^-1^ of Fe_2_O_3_ NPs and all concentrations of bulk Fe_2_O_3_ positively impacted chlorophyll and carotenoid levels. Both nano and bulk Fe_2_O_3_ supplements stimulated H_2_O_2_ production and subsequently enhanced enzymatic antioxidant activity. The use of 50 mg L^-1^ of Fe_2_O_3_ NPs resulted in the highest flavonoid content and non-enzymatic antioxidant activity. Meanwhile, the highest essential oil content and yield was achieved by the application of 50 and/or 100 mg L^-1^ Fe_2_O_3_ NPs. The addition of low concentration of Fe_2_O_3_ NPs (50 mg L^-1^) resulted in a significant increase in the concentration of geranial, while a higher supply of Fe_2_O_3_ NPs (200 mg L^-1^) significantly decreased the percentage of neral in the essential oil. Overall, the application of Fe_2_O_3_ NPs demonstrated significant potential for increased biomass, enhanced yield, essential oil production, and phytochemical attributes. The findings highlight the versatility of Fe_2_O_3_ NPs at optimal concentrations, acting as both a nano-fertilizer and a nano-inducer, promoting the production and accumulation of valuable secondary metabolites in plants.

## 1 Introduction

*Dracocephalum kotschyi* Boiss., a perennial herb belonging to the Lamiaceae, is a valuable medicinal plant endemic to Iran. It grows in cold and high-altitude areas (Sadraei et al., 2022). This herb is renowned for its natural food flavoring properties and has been traditionally used to treat various diseases including rheumatism, gastrointestinal disorders, and asthma (Hosseini-Sharifabad et al., 2021). Numerous studies have demonstrated the antioxidant, antibacterial, anticancer, antidiabetic, anti-hyperlipidemia, anti-inflammatory, antispasmodic, and immunomodulatory properties of *D. kotschyi* (Hosseini-Sharifabad et al., 2021). The essential oil of *D. kotschyi* is rich in terpenes such as 1,8-cineole, carvone, carvacrol, (*E*)-caryophyllene, neral, menthone, limonene, and α-terpineol (Sonboli et al., 2019). Currently, there is a growing demand for the wild collection of this endemic plant to meet the needs of the food and pharmaceutical industries. However, this practice poses challenges like the extinction of landraces, the decline in genetic diversity, and unreliable herbaceous material supply. To address these issues, there is a focus on domestication and cultivation to safeguard genetic resources and ensure a sustainable supply of raw plant material (Tariq et al., 2024).

The biosynthesis of secondary metabolites in plants is influenced by various environmental factors, including climate, soil, nutrition, and ecological conditions (Mishra, 2024; Mumivand et al., 2023). Consequently, potential changes in environmental conditions can pose challenges for the growth and development of certain medicinal species (Pant et al., 2021). The presence of active substances in plants is a result of their interaction with the environment over a long evolutionary process. The production and alterations of these substances are closely correlated and associated with environmental factors. Some substances are only synthesized under specific conditions, while the levels of certain substances may substantially increase under particular circumstances (Jadidi et al., 2023).

Iron is crucial for plant growth and ranks as the fourth most prevalent element in the Earth’s crust. Iron plays a vital role in chlorophyll and DNA biosynthesis, photosynthesis, respiration, nitrogen fixation, energy transfer, and the activity of certain enzymes such as cytochrome oxidase, peroxidase, and catalase (Merinero et al., 2022). Iron-sulfur proteins are involved in metabolic processes like photosynthesis, sulfate reduction, respiration, and nitrogen fixation. Iron is also crucial for electron carrier production in photosystems I and II (Vallières et al., 2024). However, since plants typically absorb iron from the soil in the form of Fe^2+^, deficiency symptoms occur when a significant amount of iron remains in the insoluble Fe^3+^ form (Sreelakshmi et al., 2020). This deficiency not only leads to chlorosis but also decreases the activity of certain enzymes like catalase, ascorbate peroxidase, and peroxidase, which contain heme groups as prosthetic groups essential for plant metabolism (Majdalawieh et al., 2023). Antioxidant enzymes like catalase, ascorbate peroxidase, and peroxidase play a protective role against reactive oxygen species (ROS) damage. These enzymes are present in different parts of the cell and are responsible for transforming O_2_^-^ into H_2_O_2_ and O_2_ as part of the detoxification process (Mumivand et al., 2021a). Iron's low solubility in oxidized form in aerobic conditions makes it the third most limiting nutrient for plant metabolism and growth (Haydar et al., 2022). Therefore, efforts should be made to mitigate iron stress (toxicity or deficiency) to avoid nutritional disorders in plants.

The utilization of chelated fertilizers, particularly for iron supplementation, can effectively address iron deficiency issues in basic soils and similar conditions. However, chelated fertilizers can be expensive and may not be suitable for all plants. Conversely, the excessive application of chemical fertilizers can cause imbalances in soil minerals, degrade soil fertility and texture, and negatively impact the ecosystem (Haydar et al., 2022). Another potential solution is the use of bio-fertilizers, but their large-scale production is challenging, and their effectiveness is dependent on pH and temperature conditions. In this regard, nanotechnology offers a promising approach, with iron oxide nanoparticles like Fe_2_O_3_ being a potential solution for iron deficiency in plants (Ahmed et al., 2023).

Nanoparticles (NPs) have diverse applications in agriculture, including genetic modification of plants, promoting growth and development, and regulating the release of agrochemicals (Grillo et al., 2021). NPs enable precise delivery and regulated release of agrochemicals and essential macromolecules necessary for plant growth and development, promoting efficient utilization and reduced environmental impact (Rajput et al., 2020). Nowadays, there is an increased interest in the utilization of metal-based NPs, including Fe_2_O_3_ NPs, in agriculture. Ferric oxide NPs are safe and environmentally friendly materials that can positively influence the biochemical composition and growth of plants. They enhance plant growth, promote the levels of medicinal and nutritional properties in plants, facilitate nutrient uptake, and increase the content of essential elements in plants through improved nutrient absorption and increased photosynthesis, which is an important metabolic pathway under the influence of iron NPs (Ahmed et al., 2023). Nemati Lafmejani et al. (2018) demonstrated the effectiveness of iron NPs applied during the flowering stage on the yield and constituents of essential oil of peppermint (*Mentha* × *piperita* L). Similarly, Ghassemi-Golezani and Abdoli (2021) investigated the effects of foliar spraying of salicylic acid and Fe_2_O_3_ NPs on *Trachyspermum ammi* (L.) Sprague at two stages (7-leaf and flowering stages) and found that the combination of NPs and salicylic acid could enhance the essential oil content. Due to their small size, Fe_2_O_3_ NPs can enter plant cells via iron channels, aquaporins, or binding to carrier proteins (Sharma et al., 2023). Fe_2_O_3_ NPs have shown positive effects on antioxidant enzyme activity in *Triticum aestivum* L., as well as enhanced antioxidant activity (Alshegaihi et al., 2024). In the case of *D. kotschyi*, Fe_2_O_3_ NPs at the level of 75 mg L^−1^ after 24 h treatment have been observed to stimulate the production of phenolic compounds in the hairy roots (Nourozi et al., 2019). Similarly, in *Hyoscyamus reticulatus* L., treatment with Fe_2_O_3_ NPs resulted in a substantial increase in the production of scopolamine and hyoscyamine compared with controls (Moharrami et al., 2017).

*D. kotschyi*, classified as a vulnerable species in Iran according to the IUCN grouping criteria (Moradi et al., 2020), is facing challenges due to overcollection from its natural habitats. To address this issue, there is an increasing inclination towards cultivating this plant within agricultural systems. However, there has been relatively limited research and development focused on issues in cultivation of this species. Particularly, there is no information regarding the effects of both nano and bulk forms of iron oxide, which belong to the new generation of elicitors, on the plant growth and essential oil composition of *D. kotschyi*. Considering that both nano and bulk forms of iron oxide are extensively used in various industries, this research was aimed to investigate their impact on the growth, yield, total phenolic and flavonoid content, essential oil production, and antioxidant capacity of *D. kotschyi*.

## 2 Materials and methods

### 2.1 Fe_2_O_3_ NPs characterization

**Figure 1:**
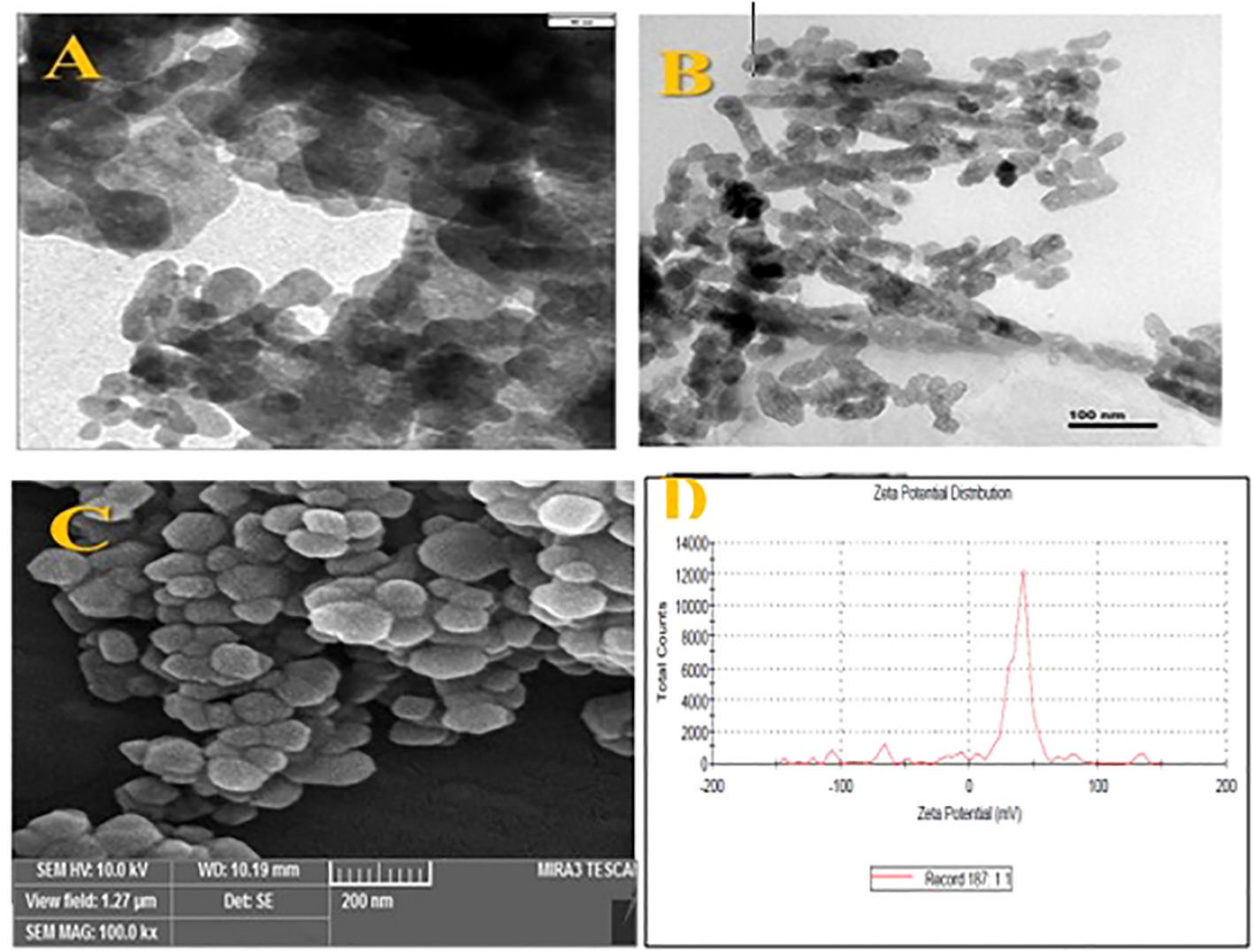
The TEM images **(A, B)**, SEM images **(C)** and zeta potential graph **(D)** of Fe2O3 NPs used in this study.

Fe_2_O_3_ NPs obtained from Nanosany Company, Ltd., Iran, were subjected to characterization using scanning electron microscopy (SEM) and transmission electron microscopy (TEM) (**Figure 1**). The Zetasizer Nano ZS instrument was used to determine the zeta potential and diameter values of the Fe_2_O_3_ (S1).

### 2.2 Plant material and greenhouse experiments

For the greenhouse experiments, *D. kotschyi* seeds were acquired from Company of Pakan Bazr in Isfahan, Iran. The seeds underwent surface sterilization with 2% NaOCl and were rinsed with distilled water. Subsequently, the sterilized seeds were sown in seedling trays containing autoclaved coco peat for germination with two seeds per cell, in November 2022. Seedling trays were placed in research greenhouses of the Horticultural Science Department, Lorestan University, Khorramabad, Lorestan Province, Iran. Seedlings with 6-8 leaves were transplanted into individual pots (10 cm in diameter and 12 cm in height). The substrate used for the pots was prepared by combining sand, agricultural soil, and manure (3:1:1). The results of analysis of pot soil can be found in **Supplementary Table S2**. Foliar application of Fe_2_O_3_ included: control (sprayed with distilled water), 50 mg L^-1^ bulk Fe_2_O_3_, 100 mg L^-1^ bulk Fe_2_O_3_, 200 mg L^-1^ bulk Fe_2_O_3_, 50 mg L^-1^ Fe_2_O_3_ NPs, 100 mg L^-1^ Fe_2_O_3_ NPs, and 200 mg L^-1^ Fe_2_O_3_ NPs. The experiment was conducted following a completely randomized design (CRD) with 3 replications (5 pots per each replication). The proper concentration range of foliar spray was selected based on both a comprehensive literature survey and a pre-experiment (Rizwan et al., 2018; Hassanpouraghdam et al., 2022; Moradbeygi et al., 2020) and a pilot experimental study. The applications were performed every two weeks until harvesting, resulting in a total of six sprays. The first spray was administered on March 7^th^ 2023, and the final spray was conducted on May 16^th^ 2023. During the entire five-months period of plant phenology, effective measures were implemented to control weeds, pests, and diseases in the greenhouse. The greenhouse itself was oriented in a north-south direction. The average temperatures during the night and day were maintained at around 20 and 30°C, respectively. The relative humidity levels ranged from 55 to 85%. Inside the greenhouse, the photosynthetically active photon flux density was approximately 600 ± 100 μmol quanta m^–2^ s^–1^.

### 2.3 Growth parameters

At the full flowering stage of growth, various growth parameters were measured, including height of plant (cm), diameter of stem (mm), length of internode (mm), length and width of leaf (mm), and length of inflorescence (cm). Then, plants were harvested and the fresh weight of aerial parts was measured. Plant aerial parts were oven-dried at 50°C for determination of dry weight. Finally, leaves were separated from stems and dry weights of stem, dry weights of leaf, as well as leaf/stem ratio were recorded.

### 2.4 Photosynthetic pigments and relative water content

To measure the contents of carotenoids, chlorophyll b, and chlorophyll a, the Lichtenthaler (1987) method was employed. Fresh frozen leaf samples weighing 0.25 g were extracted in a 10 mL solution of 80% acetone. The obtained extract was subsequently centrifuged for 15 min at 10,000 rpm. The absorbance of the resulting supernatant was recorded at wavelengths of 663, 645, and 470 nm.

The relative water content (RWC) of leaf samples was assessed using the Ferrat and Lova (1999) method. Ten fully expanded leaves were collected and promptly weighed to assess the fresh weight (FW). The selected leaves were then immersed in distilled water for 24 h at a temperature of 25°C to attain their saturated weight (SW). After the absorption period, the leaf samples were dried for 48 h at 85°C to measure the dry weight (DW). The RWC was measured according to the formula provided and repeated three times.


RWC=(FW−DW)/(SW−DW)×100

### 2.5 Total phenols and flavonoids content and antioxidant activity

To prepare the herbal extract, 100 mg samples from each treatment were separately mixed with 2 mL of an 80% methanol solution and left to incubate in a dark room for 16 h. Following centrifugation for 20 min at 14,000 × g, the clarified supernatant was subsequently filtered.

The content of total phenolics was measured using the Folin-Ciocalteau reagent (McDonald et al., 2001). In brief, a 0.4 mL diluted extract of the leaf was combined with 1.6 mL of sodium carbonate solution and 2 mL of the Folin-Ciocalteau reagent. Following incubation at room temperature for 30 min, the absorbance was read at 760 nm. The content of total phenolics was expressed as mg of gallic acid equivalents (GAE) per g dry weight of plant (mg GAE/g DW).

To evaluate the flavonoid content, the AlCl_3_ colorimetric method was employed (Hang et al., 2002). Briefly, 2 mL of the diluted extract was mixed with 2% aluminum chloride (2 mL) and stored for 15 min at room temperature. The resulting mixture was then read for absorbance at 415 nm. The total content of flavonoid was expressed as mg of rutin per g of plant dry weight (mg rutin/g DW).

The antioxidant activity of extracts was measured by FRAP assay according to Benzie and Strain (1996). In test tubes, 0.02 mL of the extract and 0.18 mL of the FRAP reagent were combined and incubated for 30 min at 30°C. The absorbance was read at 593 nm by using a spectrophotometer (Shimadzu, model UV-1700, Japan).

### 2.6 Fe concentration

The concentration of Fe was measured according to the method described by Bao (2000) with slight modifications. Approximately 1 g of the sample was subjected to ashing in a muffle furnace at a temperature of 500°C for 3 h. After the sample had cooled down, the resulting ash was dissolved in 10 mL of 2-normal hydrochloric acid and transferred to a 50 mL flask. The concentration of Fe was then determined using Atomic Absorption Spectrometry (model Agilent 240FS AA, USA). The nutrient element concentration was expressed as mg kg^-1^ of plant dry weight (DW).

### 2.7 Phenylalanine ammonia-lyase activity

A 0.5 g of fresh leaves was powdered in 5 mL of a phosphate buffer (0.05 M, pH 7.8) containing 1% PVP and 1mM EDTA. The resulting mixture was then centrifuged at 12,000×g (20 min, 4°C). The supernatant was collected for the measuring ascorbate peroxidase (APX) and phenylalanine ammonia-lyase (PAL) activity. The activity of PAL was measured using a modified assay based on a method described by Whetten and Sederoff (1992). In brief, the reaction mixture comprised 1 mM L-phenylalanine, the enzyme extract, and 50 mM Tris-HCl (pH 8.8) (total volume of 1.2 mL). The reaction proceeded for 30 to 60 min at 30°C and was stopped by adding 0.5 mL of 4N HCl. After vortexing with 2 mL of toluene, the mixture underwent centrifugation for 5 min at 750 g. The absorbance of the cinnamic acid retrieved in the toluene phase was read at 290 nm.

### 2.8 Enzymatic antioxidants activity

The activity of APX was assessed according to the Nakano and Asada (1981) method. The enzyme extract was combined with a reaction mixture consisting of ascorbic acid (AA) (0.5 mM), H_2_O_2_ (0.1 mM), EDTA (0.1 mM), and phosphate-potassium buffer (50 mM, pH 7.0). The absorbance was read at 290 nm.

For the extraction of peroxidase (POD) and catalase (CAT), leaf samples (0.5 g fresh leaves) were extracted with 2 mL of Tris buffer (0.1 mol/L, pH 8) consisting of 1 mM EDTA and 0.05 g PVP. The mixture was centrifuged at 13,000 g (15 min at 4°C). A 0.05 mL of the supernatant was added to 0.4 mL of extraction buffer (50 mM, pH 7.0) containing 0.4 ml H_2_O_2_ (12.5 mM). The activity of CAT was monitored every 10 sec for 2 min at 240 nm (Gallego et al., 1996).

The activity of POD was measured by observing the oxidation of guaiacol catalyzed by peroxidase enzyme (Zhang et al., 1995). The reaction mixture contained 50 µL of 30% H_2_O_2_, 50 µL of crude extract, and 50 µL of 0.05 M guaiacol in 1200 µL of phosphate buffer (50 mM, pH 7.0). The change in absorbance was recorded at 470 nm every 10 sec.

### 2.9 Hydrogen peroxide

The level of hydrogen peroxide (H_2_O_2_) was measured according to the method of Velikova et al. (2000). Fresh leaves weighing 0.5 g were powdered with 5 mL of trichloroacetic acid (TCA) (0.1% w/v). After centrifugation for 15 min at 10,000 g, 0.5 mL of the resulting supernatant was combined with 0.5 mL of potassium phosphate buffer (pH 7.0) and 2 mL of potassium iodide. The absorbance of samples was read at 390 nm.

### 2.10 Essential oil isolation

A quantity of 25 g of dried leaves was individually subjected to hydrodistillation utilizing a Clevenger apparatus for a period of 3 h. The essential oil obtained was collected in a glass vial. The content of essential oil was measured and expressed as mg per 100 g of dry plant (w/w). The yield of essential oil was obtained by multiplying of the content of essential oil with the dry weight of the aerial parts (g plant^-1^) (Taheri-Garavand et al., 2021).

### 2.11 Essential oil analysis

The separation of essential oil constituents was achieved using GC-MS with a capillary column of BP-5 fused-silica. The carrier gas was helium, with the temperature of injector and interface set at 280 and 260°C, respectively. The electron impact ionization was 70 eV, and the split ratio was 1/20. The mass spectrum was recorded in the range of 35–450 amu. The oven temperature was increased from 55 to 285°C at a rate of 4°C per min and for 15 min held isothermally (Beiranvandi et al., 2022).

GC-FID analysis was performed using a Thermoquest Finnigan equipped with a BP-5 capillary column (30 m length, 0.22 mm inner diameter, and 0.25 μm film thickness) and FID detector. The oven temperature was set as same as the GC-MS analysis. The temperatures of detector and injector were maintained at 300 and 250°C, respectively. Helium was utilized as the carrier gas (flow rate of 1 mL/min). Retention indices (RI) of essential oil components were measured using a *n*-alkanes series (C6-C40) injected into the GC system. The identification of constituents was performed by (1) mass spectra comparison with an internal library (WILEY 275 and NIST 17), (2) the calculated RI compared with those reported in Adams book, and (3) the retention time compared with those of available standards (Mumivand et al., 2021c)

### 2.12 Data analysis

The data obtained were analyzed using SAS statistical software based on the experimental design employed in the study. After analysis of variance (ANOVA), mean comparison between all treatments was done using the LSD test at a 0.05% significance level.

## 3 Results

### 3.1 Biomass and yield attributes

The findings of the study revealed significant effects of foliar spraying with Fe_2_O_3_ NPs treatment on various growth parameters of *D. kotschyi*. The plant height, inflorescence length, leaf length, stem diameter, internode length, leaf dry weight, stem dry weight, and ratio of leaf/stem were all influenced by the application of Fe_2_O_3_ (**Supplementary Table S3**). The highest plant height was observed with the application of 200 mg L^-1^ of Fe_2_O_3_ NPs, resulting in a 26.5% increase compared with control plants. Additionally, plants sprayed with 100 mg L^-1^ of bulk Fe_2_O_3_ showed a 9.036% increase in plant height compared with the control. The inflorescence length was elongated by the application of Fe_2_O_3_ NPs up to the level of 100 mg L^-1^, and bulk Fe_2_O_3_ up to 50 mg L^-1^. However, higher concentrations had a negative impact on the inflorescence length. The length of leaves showed an increase with the foliar spraying of both nano and bulk forms of Fe_2_O_3_, with the highest value recorded at a concentration of 200 mg L^-1^ of Fe_2_O_3_ NPs (25.07 mm). The maximum stem diameter was observed with the application of 50 mg L^-1^ of Fe_2_O_3_ NPs, while different concentrations of bulk Fe_2_O_3_ did not significantly affect stem diameter. Internode length was increased by the application of Fe_2_O_3_ NPs up to the level of 200 mg L^-1^, and bulk Fe_2_O_3_ up to 100 mg L^-1^. The highest increase in internode length (67.63% compared with the control) was obtained with the application of 200 mg L^-1^ of Fe_2_O_3_ NPs. Regarding biomass, the foliar spray of 100 and/or 200 mg L^-1^ of Fe_2_O_3_ NPs led to the highest dry weight of leaf (3.39 g), while the highest dry weight of stem was observed in plants sprayed with 50 and/or 200 mg L^-1^ of bulk Fe_2_O_3_ (2.22 and 2.46 g, respectively). The highest leaf/stem ratio was recorded by the application of Fe_2_O_3_ NPs at the level of 100 mg L^-1^, followed by the level of 200 mg L^-1^ (2.87 and 1.78, respectively) (**Table 1**).

**Table 1:** Mean comparison of the effects of foliar application of Fe_2_O_3_NPs and bulk Fe_2_O_3_ on biomass and yield attributes of *D. kotschyi*.

Fe_2_O_3_ Application (mg L^-1^)	Plant height (cm)	Inflorescence length (cm)	Leaf length (mm)	Stem diameter (mm)	Internode length (mm)	Leaf dry weight (g)	Stem dry weight (g)	Leaf/Stem ratio
Control	41.5^d^	8.9^d^	19.9^d^	1.74^bc^	22^e^	2.3^b^	1.6^bc^	1.4^bc^
Nano 50	40.5^d^	10.2^cd^	21.6^cd^	2.3^a^	28.8^cd^	1.4^c^	0.9^d^	1.1^cd^
Nano 100	42.5^cd^	14.6^ab^	24.1^ab^	1.99^ab^	32.8^b^	3.4^a^	1.2^cd^	2.9^a^
Nano 200	52.5^a^	10.8^c^	25.1^a^	1.9^bc^	37^a^	3.4^a^	1.9^ab^	1.9^b^
Bulk 50	36^e^	15.5^a^	23.1^abc^	1.91^b^	29.7^c^	2.3^b^	2.2^a^	1.1^d^
Bulk 100	45.3^b^	13.2^b^	22.4^bc^	1.83^bc^	34^b^	2.4^b^	1.6^bc^	1.5^bc^
Bulk 200	44.5^bc^	6.3^e^	22.4^bc^	1.6^c^	27.7^d^	2.5^b^	2.5^a^	1^d^

Means with the same letters in each column do not have a significant difference at the 1% probability level based on the Duncan test.

### 3.2 RWC and photosynthetic pigments

The analysis of variance (**Supplementary Table S4**) revealed a significant effect of foliar application of Fe_2_O_3_ on the RWC, carotenoid, total chlorophyll, chlorophyll b, and chlorophyll a levels in *D. kotschyi* (*p* ≤ 0.01). The spray of bulk Fe_2_O_3_ had a positive impact on leaf RWC, with the highest value observed at a concentration of 200 mg L^-1^ (86.8%). In contrast, there was no significant difference in leaf RWC with any of the Fe_2_O_3_ NPs concentrations. Foliar application of Fe_2_O_3_ NPs at a concentration of 200 mg L^−1^ resulted in increased contents of total chlorophyll, chlorophyll a, and carotenoids. However, the highest values of total chlorophyll and chlorophyll a were observed in plants exposed to 100 mg L^−1^ of bulk Fe_2_O_3_, which had no significant difference with treatments of 200 mg L^-1^ of Fe_2_O_3_ NPs as well as all other concentrations of bulk Fe_2_O_3_. The highest content of carotenoids was obtained with the application of 200 mg L^−1^ of Fe_2_O_3_ NPs and all concentrations of bulk Fe_2_O_3_ (**Table 2**).

**Table 2:** Mean comparison of the effects of foliar application of Fe_2_O_3_NPs and bulk Fe_2_O_3_ on biochemical traits and Fe concentration of *D. kotschyi*.

Fe_2_O_3_ Application (mg L^-1^)	Relative water content (%)	Chlorophyll a (mg g^-1^ FW)	Chlorophyll b (mg g^-1^ FW)	Total chlorophyll (mg g^-1^ FW)	Carotenoid (mg g^-1^ FW)	Total phenol (mg GAE g^-1^ DW)	Flavonoids (mg rutin g^-1^ DW)	FRAP assay (µmol Fe^+2^ g^-1^ DW)	Fe concentration (mg g^-1^ DW)
Control	72.4^c^	0.8^b^	0.24^cd^	1.08^b^	8 ^b^	0.29^a^	48.5^b^	2.6^c^	88^d^
Nano 50	71.8^c^	0.9^b^	0.26^c^	1.02^b^	7.4^b^	0.27^cd^	55^a^	4^a^	153^c^
Nano 100	74^c^	0.7^c^	0.20^d^	0.94^b^	7^b^	0.275^c^	43.8^c^	2.6^c^	258.1^b^
Nano 200	75.4^c^	1.02^a^	0.26^c^	1.37^a^	10.5^a^	0.28^ab^	43.2^c^	2.5^c^	477.1^a^
Bulk 50	76^c^	1^a^	0.32^b^	1.30^a^	9.3^a^	0.26^e^	53.5^a^	3.3^b^	98.1^d^
Bulk 100	81.4^b^	1.1^a^	0.37^a^	1.43^a^	10.3^a^	0.265^ed^	33.2^e^	3.4^b^	101.5^d^
Bulk 200	86.8^a^	1.1^a^	0.23^cd^	1.41^a^	10 ^a^	0.276^bc^	37.1^d^	3.7^ab^	170.5^c^

Means with the same letters in each column do not have a significant difference at the 1% probability level based on the Duncan test.

### 3.3 Total phenolic and flavonoid contents and antioxidant capacity

The application of Fe_2_O_3_, whether in nano or bulk form, had a significant impact on the contents of total phenolics and flavonoids, as well as non-enzymatic antioxidant activity in the leaves of *D. kotschyi* (*p* ≤ 0.01) (**Supplementary Table S2**). In terms of total phenolics, the foliar application of Fe_2_O_3_ in both nano and bulk forms (except for 200 mg L^−1^ of Fe_2_O_3_ NPs) resulted in a significant decrease in level compared with the control. However, spraying with 50 mg L^−1^ of Fe_2_O_3_ in both forms resulted in a significant increase in the total flavonoids content of the samples compared with the control. Conversely, higher concentrations of Fe_2_O_3_ led to a significant decrease in the total flavonoids content. The maximum non-enzymatic antioxidant activity was observed with the application of 50 mg L^−1^ of Fe_2_O_3_ NPs (3.97 μmol Fe^2+^ g^-1^ DW) and/or 200 mg L^−1^ of bulk Fe_2_O_3_ (3.71 μmol Fe^2+^ g^-1^ DW) (**Table 2**).

### 3.4 Fe concentration

The foliar spraying of Fe_2_O_3_ had a significant influence (*p* ≤ 0.01) on the Fe content in the leaves of *D. kotschyi*, as indicated in **Supplementary Table S2**. The results revealed that increasing concentrations of Fe_2_O_3_ NPs were associated with an increase of the Fe content of the leaves. The highest Fe content was observed in plants sprayed with Fe_2_O_3_ NPs at the level of 200 mg L^−1^, reaching a value of 477.10 mg g^-1^ DW. Furthermore, the application of 200 mg L^−1^ of bulk Fe_2_O_3_ also resulted in an increase of the Fe content compared with the control.

### 3.5 PAL activity

**Figure 2:**
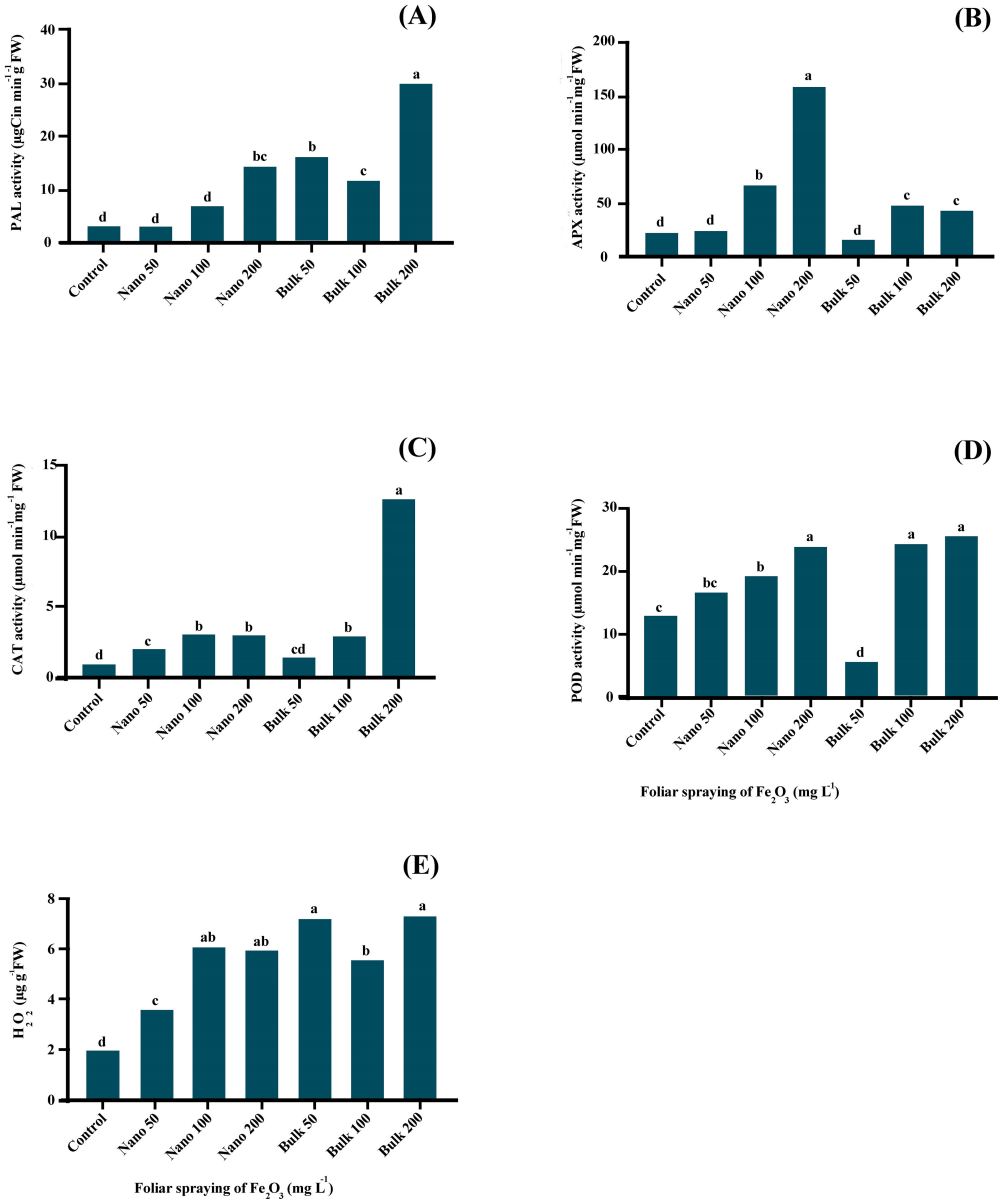
The effect of foliar application of Fe_2_O_3_NPs and bulk Fe_2_O_3_ on PAL activity **(A)**, APX **(B)**, CAT **(C)**, POD **(D)** activity and H_2_O_2_ content **(E)** of *D. kotschyi*. (Columns with different letters in each chart have a significant difference at the 1% probability level based on the Duncan test).

The current investigation revealed a significant effect (*p* ≤ 0.01) of Fe_2_O_3_ on the activity of the PAL enzyme, as indicated in **Supplementary Table S5**. The foliar spraying of bulk Fe_2_O_3_ significantly increased PAL activity in the leaf samples. The highest PAL activity was obtained in the leaves sprayed with 200 mg L^−1^ of bulk Fe_2_O_3_, reaching a value of 29.711 μg Cin min^-1^g^-1^ FW. On the other hand, the foliar spray of Fe_2_O_3_ NPs only increased PAL activity at the 200 mg L^−1^ treatment (**Figure 2A**).

### 3.6 Antioxidant enzymes activity and H_2_O_2_ content

The foliar application of Fe_2_O_3_ had a significant impact (*p* ≤ 0.01) on the activity of antioxidant enzymes involved in free radical scavenging, namely APX, CAT, and POD, as well as the H_2_O_2_ content. APX activity significantly increased when plants were exposed to concentrations of 100 and/or 200 mg L^−1^ of Fe_2_O_3_ NPs, with the highest boost observed at the level of 200 mg L^−1^, which was 6.33 times higher than the control. On the other hand, the application of bulk Fe_2_O_3_ led to increase APX activity at the levels of 100 and/or 200 mg L^−1^ (**Figure 2B**). Both nano and bulk forms of Fe_2_O_3_ led to an increase in CAT activity in the leaves. The highest CAT activity was obtained in plants sprayed with 200 mg L^−1^ of bulk Fe_2_O_3_ (12.6904 μmol min^−1^ g^−1^ FW), while the lowest activity was found in untreated plants (0.9259 μmol min^−1^ g^−1^ FW) (**Figure 2C**). The concentration of Fe_2_O_3_ NPs showed a linear induction of POD activity. However, significant increases in POD activity were obtained with the application of higher concentrations of bulk Fe_2_O_3_ (100 and/or 200 mg L^−1^). The highest POD activity was recorded at the concentrations of 100 and/or 200 mg L^−1^ of bulk Fe_2_O_3_, as well as 200 mg L^−1^ of Fe_2_O_3_ NPs (**Figure 2D**). Foliar application of both nano and bulk forms of Fe_2_O_3_ induced the accumulation of H_2_O_2_ in plant leaves. The highest H_2_O_2_ content was obtained in plants sprayed with 200 mg L^−1^ of bulk Fe_2_O_3_, although there was no significant difference compared with 50 mg L^−1^ of bulk Fe_2_O_3_, as well as 100 and/or 200 mg L^−1^ of Fe_2_O_3_ NPs (**Figure 2E**).

### 3.7 Essential oil content and yield

**Figure 3:**
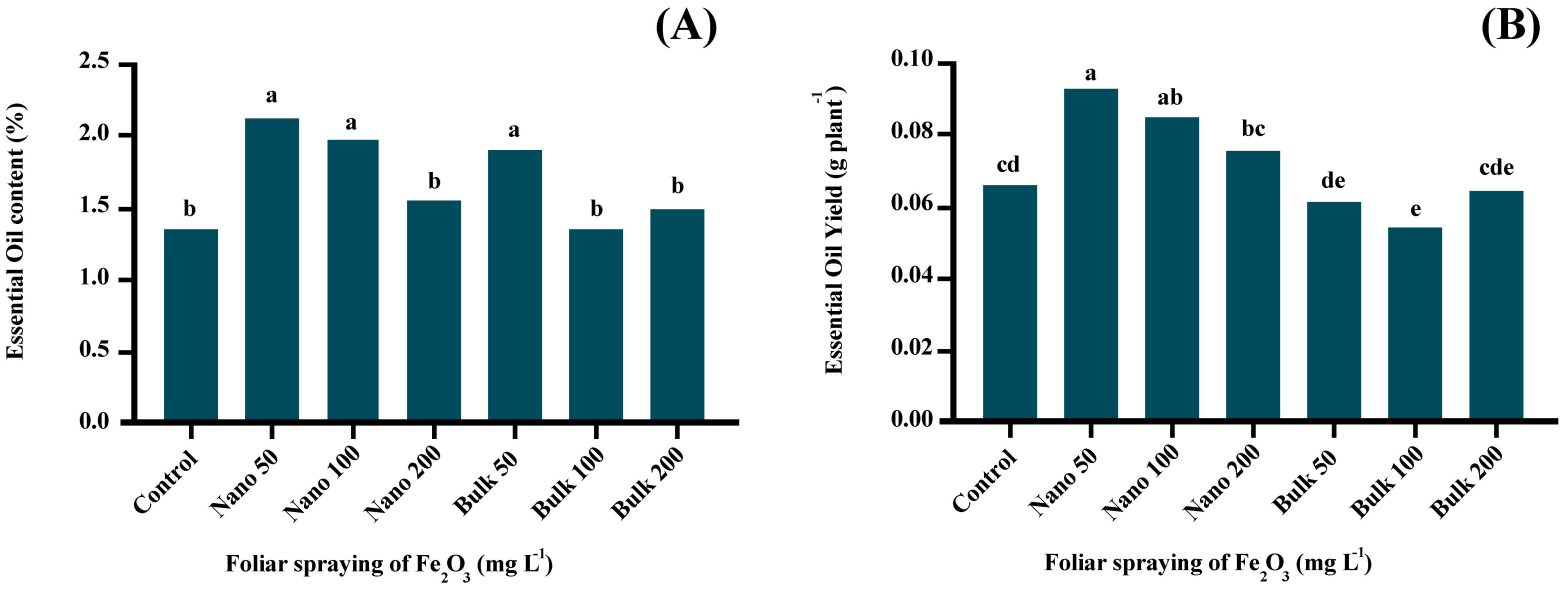
The effect of foliar application of Fe_2_O_3_NPs and bulk Fe_2_O_3_ on the essential oil content **(A)** and essential oil yield **(B)**. (Columns with different letters in each chart have a significant difference at the 1% probability level based on the Duncan test).

The analysis of variance demonstrated significant effects (*p* ≤ 0.01) of foliar application of Fe_2_O_3_ on the content and yield of *D. kotschyi* essential oil. The application of 50 mg L^−1^ of both nano and bulk forms of Fe_2_O_3_, as well as 100 mg L^−1^ of Fe_2_O_3_ NPs, led to a significant enhancement in the essential oil content. The highest percentage of essential oils (2.138%) was recorded with foliar application of 50 mg L^−1^ of Fe_2_O_3_ NPs, which showed no significant difference compared with 100 mg L^−1^ of Fe_2_O_3_ NPs and 50 mg L^−1^ of bulk Fe_2_O_3_ treatments. However, as the concentration of NPs and bulk forms of Fe_2_O_3_ increased, the essential oil content declined. (**Figure 3A**). Furthermore, the essential oil yield was significantly increased in plants treated with Fe_2_O_3_ NPs at the level of 50 mg L^−1^ compared with the control plants. The highest essential oil yield (0.0917 g/plant) was obtained from *D. kotschyi* plants treated with 50 mg L^−1^ of Fe_2_O_3_ NPs, which showed no significant difference compared with the 100 mg L^−1^ of Fe_2_O_3_ NPs treatment. However, higher levels of Fe_2_O_3_ NPs resulted in a decline in the essential oil yield. Different concentrations of bulk Fe_2_O_3_ did not make any significant change in the essential oil yield compared with the control (**Figure 3B**).

### 3.8 Essential oil composition

The GC-MS and GC-FID analysis of *D. kotschyi* essential oil resulted in the identification of 22 constituents. Among these, the main components were found to be geranial (up to 34.005%) and neral (up to 23.18%), which are isomers of citral. Additionally, remarkable amounts of geranyl acetate (up to 10.708%), methyl geranate (up to 7.347%), δ3-carene (up to 6.52%), and *α*-pinene (up to 5.34%) were also present in the essential oil. Notably, the variance analysis revealed that the foliar spraying of Fe_2_O_3_ had a significant influence on various components of the essential oil, including δ3-carene, *cis*-sabinene hydrate, *α*-campholenal, *E*-2,6-nonadien-1-ol, *cis*-verbenol, *iso*-pulegone, 1,3,4-trimethyl-3-cyclohexenyl-1-carboxaldehyde, neral, geranial, methyl geranate, geranyl acetate, (*E*)-caryophyllene, aromandendrene, and *β*-gurjunene (**Supplementary Table S6**).

The mean comparison of the effect of Fe_2_O_3_ foliar spraying on the essential oil components showed that the spraying of 50 mg L^-1^ of Fe_2_O_3_ NPs significantly increased the level of geranial, the most important component of the essential oil, by 10.08% compared with the control. The control plants exhibited the highest percentage of neral (23.18%), while the lowest value was obtained in the treatment with 200 mg L^−1^ of Fe_2_O_3_ NPs (19.346%). However, no significant difference was noticed between the control and other plants treated with both nano and bulk forms of Fe_2_O_3_ in terms of neral percentage. The highest percentage of geranyl acetate was obtained with foliar spraying of Fe_2_O_3_ NPs at the level of 100 mg L^−1^, but it did not significantly differ from 50 mg L^−1^ of Fe_2_O_3_ NPs, 200 mg L^−1^ of bulk Fe_2_O_3_, and control treatments. The content of δ3-carene was significantly increased with the foliar application of 200 mg L^−1^ of Fe_2_O_3_ NPs, as well as 50 and/or 100 mg L^−1^ of bulk Fe_2_O_3_ compared with the control. The highest percentage of δ3-carene was obtained in plants sprayed with Fe_2_O_3_ NPs at the level of 200 mg L^−1^, which was 68.6% higher than the control. The spraying of 50 and/or 100 mg L^−1^ of Fe_2_O_3_ NPs significantly decreased the percentage of methyl geranate compared with the control, with the highest value observed in 50 mg L^−1^ of bulk Fe_2_O_3_. The highest percentage of linalool and *iso*-pulegone (0.974 and 0.55%, respectively) was observed with the application of 200 mg L^−1^ of Fe_2_O_3_ NPs. On the other hand, foliar spraying of Fe_2_O_3_ NPs led to a significant decline in the level of 1,3,4-trimethyl-3-cyclohexenyl-1-carboxaldehyde, with the lowest percentage of this compound obtained with the highest level of Fe_2_O_3_ NPs. (*E*)-Caryophyllene was only detected in 50 and/or 100 mg L^−1^ of Fe_2_O_3_ NPs (**Table 3**).

**Table 3:** Mean comparison of the effects of foliar application of Fe_2_O_3_NPs and bulk Fe_2_O_3_ on *D. kotschyi* essential oil chemical composition.

NO	Constituents	RI^І^	LIT RI^ІІ^	Treatments						
				control	Nano 50	Nano 100	Nano 200	Bulk 50	Bulk 100	Bulk 200
1	*α*-Pinene	924	931	5.4^a^	4.6^a^	4.7^a^	5^a^	5.3^a^	5^a^	4.7^a^
2	Camphene	941	943	0.1^a^	0.07^a^	0.07^a^	0.06^a^	0.08^a^	0.07^a^	0.1^a^
3	Sabinene	964	964	0.9^a^	0.8^a^	0.8^a^	0.9^a^	0.9^a^	1 ^a^	0.9^a^
4	*β*-Myrcene	978	979	2.5^a^	2^a^	1.9^a^	2.2^a^	2.5^a^	2.5^a^	2.3^a^
5	*α*-Phellandrene	996	997	0.5^a^	0.4^a^	0.5^a^	0.3^a^	0.5^a^	0.4^a^	0.3^a^
6	δ3-Carene	1020	1008	6.5^cd^	6.9^c^	6.4^cd^	11^a^	8.6^b^	9.5^b^	5.8^d^
7	*cis*-Sabinene hydrate	1063	1068	0.5^abc^	0.27^d^	0.32^cd^	0.49^ab^	0.49^ab^	0.38^bcd^	0.55^a^
8	Terpinolene	1076	1078	0.4^a^	0.32^a^	0.4^a^	0.38^a^	0.38^a^	0.32^a^	0.4^a^
9	Linalool	1094.	1095	1.2^ab^	1.12^ab^	1^ab^	0.98^b^	1.3^a^	1.03^ab^	1.05^ab^
10	*α*-Campholenal	1121	1120	0.6^ab^	0.56^abc^	0.56^abc^	0.41^c^	0.59^ab^	0.46^bc^	0.7^a^
11	*E*-2,6-Nonadien-1-ol	1136	1143	0.5^ab^	0.32^d^	0.4^bc^	0.34^cd^	0.46^ab^	0.4^bc^	0.5^a^
12	*cis*-Verbenol	1143	1148	0.3^ab^	0.22^bc^	0.29^ab^	0.16^c^	0.3^ab^	0.25^abc^	0.35^a^
13	*iso*-Pulegone	1153	1157	1.2^a^	0.81^b^	0.97^ab^	0.55^c^	1.2^a^	0.98^ab^	1.05^a^
14	1,3,4-Trimethyl-3-cyclohexenyl-1- carboxaldehyde	1173	1171	2.1^a^	1.36^d^	1.57^cd^	0.97^e^	1.99^ab^	1.76^abc^	1.68^bcd^
15	*γ*-Terpineol	1196	1185	0.6 ^ab^	0.54^b^	0.58^ab^	0.71^a^	0.71^a^	0.6^ab^	0.56^ab^
16	Neral	1239	1240	23.2^a^	22.73^a^	22.02^a^	19.35^b^	21.77^a^	22.65^a^	22.81^a^
17	Geranial	1274	1264	34 ^bc^	37.44^a^	36.36^ab^	36.47^ab^	33.39^c^	34.73^abc^	34.84^abc^
18	Methyl geranate	1318	1319	7.4^ab^	5.57^c^	5.28^c^	6.27^bc^	8.78^a^	7^abc^	7.52^ab^
19	Geranyl acetate	1377	1360	10.7^ab^	12.19^ab^	13.5^a^	9.37^b^	9.01^b^	8.93^b^	12.32^ab^
20	(*E*)-Caryophyllene	1411	1417	–	0.021^b^	0.04^a^	–	–	–	–
21	Aromandendrene	1449	1439	0.06^abc^	0.11^a^	0.10^ab^	–	0.04^c^	0.06^bc^	0.08^a^
22	*β*-Gurjunene	1470	1463	0.6^cd^	0.89^ab^	1.1^a^	0.72^bc^	0.48^d^	0.71^bc^	0.56^cd^
	Total identified (%)			99.041	99.080	98.750	96.657	98.66	98.71	98.97

Means with the same letters in each row do not have a significant difference at the 1% probability level based on the Duncan test. ^І^ RI, linear retention indices on HP-5 column column, experimentally determined using homologue series of n-alkanes. ^ІІ^ LIT RI, Relative retention indices taken from Adams.

### 3.9 Fe_2_O_3_ NPs characteristics

**Figure 4:**
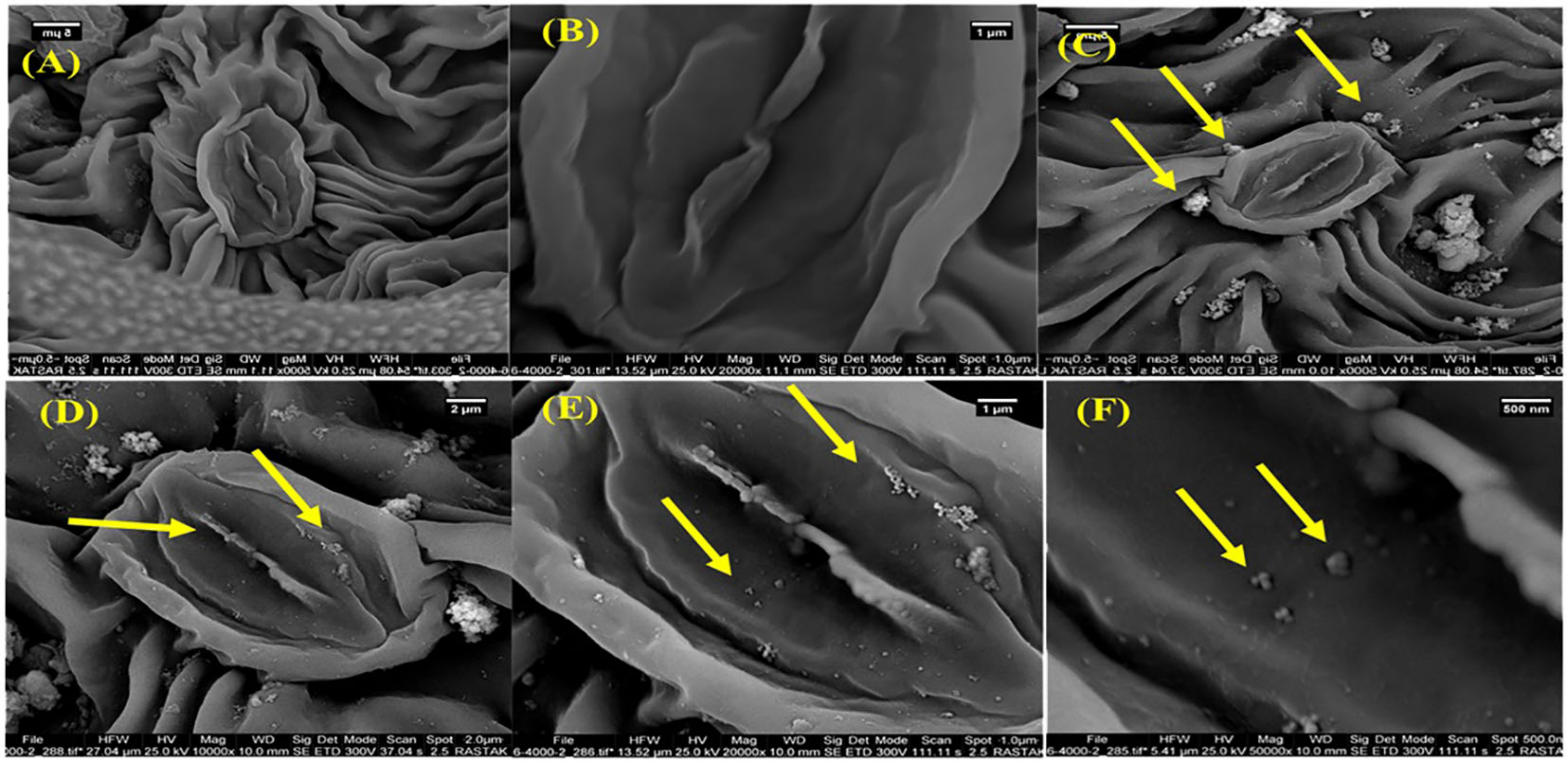
The SEM images of the leaf surface morphology of control **(A, B)** and Fe_2_O_3_ NPs-treated plants **(C–F)**. The SEM micrographs of leaves sprayed by Fe_2_O_3_NPs. Arrows show the internalization of Fe_2_O_3_ NPs inside the leaf tissues **(E, F)**.

The morphological characterization of Fe_2_O_3_ NPs synthesized for this study is detailed in **Figure 1** and **Supplementary Table S1**. TEM analyses, shown in **Figures 1A, B**, along with SEM analysis (**Figure 1C**), revealed that the Fe_2_O_3_ NPs predominantly exhibited a spherical morphology. These NPs were selected for their stability, nanoscale size, and high purity. Further experimentation investigated the interaction of Fe_2_O_3_ NPs with *D. kotschyi* plants, focusing on NPs uptake and intracellular translocation within the leaf structures. SEM analysis, illustrated in **Figure 4**, confirmed the assimilation and systemic distribution of the NPs. **Figure 4** provides a comparative analysis of foliar tissues from plants treated with a 200 mg L^-1^ concentration of Fe_2_O_3_ NPs versus an untreated control group. Specifically, segments C, D, and E of **Figure 4** highlight the deposition of NPs on the surfaces of stomatal cells, offering insights into the dynamics of NPs-plant interactions.

Dynamic Light Scattering (DLS) analysis determined the hydrodynamic size of the Fe_2_O_3_ NPs to be 203.9 nm, with a Polydispersity Index (PDI) of 0.454, indicating a relatively broad distribution of particle sizes. Zeta potential analysis revealed three distinct peaks: a primary peak at 38.5 mV, representing 75% of the sample and indicating a predominant population of positively charged NPs; a second peak at -67.5 mV, covering 4.3% of the sample; and a third peak at -7.3 mV, covering 3.2% of the sample, reflecting smaller populations of negatively charged NPs (**Figure 1D**).

## 4 Discussion

Fe_2_O_3_ NPs are known for their environmental friendliness and biocompatibility, largely due to their specific physicochemical properties, including biodegradability in natural environments. Research shows that Fe_2_O_3_ NPs, at commonly used concentrations, decompose into iron ions that are absorbed by plants and microorganisms, posing minimal environmental risks (Mousavi et al., 2021; Verma et al., 2022). Additionally, recent studies, especially those conducted since 2020, have explored the potential long-term effects of Fe_2_O_3_ NPs and found that, when applied properly, these particles present minimal environmental concerns (Zhao et al., 2023; Kumar et al., 2021).

The current study showed an increase in the biomass and yield attributes of *D. kotschyi* through the foliar application of both nano and bulk forms of Fe_2_O_3_ (**Table 1**). Previous studies have also reported growth and biomass enhancement with the application of Fe_2_O_3_ in *Oryza sativa* L (Zayed et al., 2011), *Helianthus annuus* L (Jabeen and Ahmad, 2011). and *M*. × *piperita* (Asad and Rafique, 2000). Due to the crucial role of iron in the plant photosynthesis, chlorophyll structure, and transcriptional modification, the optimal concentration of Fe_2_O_3_ improves photosynthesis and the biosynthesis of primary metabolites, which contribute to the growth-promoting effects of these supplements (Moradbeygi et al., 2020). In a study conducted by Jalal et al. (2020), it was demonstrated that the application of Fe_2_O_3_ NPs increased the metabolism, cell expansions, auxin biosynthesis, and biochemical activities of *T. aestivum*, leading to an increase in biomass and yield. Similarly, Khan et al. (2020) reported that foliar spraying of Fe NPs improved the biomass of *in vitro* cultures of *Stevia rebaudiana* (Bertoni) Bertoni. In our present study, both the bulk and nano forms of Fe_2_O_3_ supplements were found to be growth stimulants, enhancing the yield attributes of *D. kotschyi*. Notably, the nano form exhibited a greater stimulating effect on plant growth, biomass, and biofortification of tissues compared with the bulk form. This can be attributed to the unique physicochemical characteristics of nano-compounds (Neysanian et al., 2020). The different responses observed following the application of bulk or nano compounds can be attributed to variations in uptake and assimilation mechanisms, as well as the physicochemical traits of these products. Research has demonstrated that trace amounts of selenium (Se) can promote plant growth, delay senescence, and help regulation of water content under drought conditions. This is achieved by lowering ROS levels and boosting the activity of antioxidant enzymes (Rahmat et al., 2017; Djanaguiraman et al., 2018). Nano-compounds, due to their unique physicochemical characteristics like size, surface area, purity, and stability, can infiltrate the plant system through various routes including stomata and cracks on the leaf surface. Upon entering the plant, NPs can move across the plant tissues via bulk flow, diffusion, and phloem loading (Wang et al., 2023). The interaction between NPs and biomolecules may elicit different reactions in exposed plants compared with their bulk counterparts, further highlighting the importance of their physicochemical properties (Babajani et al., 2019; Sotoodehnia-Korani et al., 2020; Jafari et al., 2022). Our findings align with previous studies in *Capsicum annuum* L (Yuan et al., 2018), *Oenothera biennis* L (Asadi-Kavan et al., 2020), *Zea mays* L (Li et al., 2020). and *Nicotiana benthamiana* Domin (Cai et al., 2020).

The application of Fe_2_O_3_ in its bulk form resulted in an increase in the RWC of leaves. There are several factors that could explain the changes in plant water status following the spraying of Fe_2_O_3_. One possible reason is the structural pattern and size/density of pores in the root system, which can affect root porosity (Hatami et al., 2019). Additionally, the regulation of aquaporin expression, which influences root hydraulic conductivity and water permeability, may play a role (Yang et al., 2018). Fe_2_O_3_ application may also lead to the elicitation of antioxidant constituents and osmolytes in cells, enhancing their ability to scavenge ROS, thereby improving membrane stability and water uptake (Zhang et al., 2023). Similar changes in leaf water status have been reported in various plant species following exposure to Fe sources, including *Melissa officinalis* L (Mohasseli et al., 2020), *Eucalyptus tereticornis* Sm. (Singh et al., 2021b), and *Phaseolus vulgaris* L (Koleva et al., 2022). Previous research has recorded Se-induced changes in various photosynthesis-related traits across different plant species, such as *Solanum tuberosum* L (Germ et al., 2007). and *Ocimum basilicum* L (Ardebili et al., 2015).

The application of 200 mg L^-1^ of Fe_2_O_3_ NPs and all concentrations of bulk Fe_2_O_3_ resulted in an increase in photosynthetic pigments. This could be attributed to the significant role of Fe in chlorophyll biosynthesis and chloroplast functions (Roosta et al., 2017). According to Tombuloglu et al. (2019), the application of Fe_2_O_3_ NPs also enhances chlorophyll level of plant by up-regulating photosynthetic marker genes. Furthermore, Fe has a catalytic effect on light-activated enzymes in leaves, potentially increasing the efficiency of energy capture of the reaction center of PSII. This leads to an increase in the level of photosynthetic pigments and overall photosynthetic performance (Zhang et al., 2017). Consequently, foliar spraying of Fe_2_O_3_ enhances plant growth and yield by promoting nutrient accumulation, improving water status, and enhancing photosynthesis (Garza-Alonso et al., 2023). Similar results regarding chlorophyll levels have been observed in *Medicago sativa* L (Kim et al., 2019). and *Arachis hypogaea* L (Rui et al., 2016). when exposed to Fe NPs.

Flavonoids possess antioxidant properties and play a crucial role in regulating enzymatic activity and the production of primary metabolites. The hydroxyl groups present in the structure of flavonoids enable them to function as scavengers of free radicals (Kiumarzi et al., 2022). The findings of the current study demonstrate the beneficial influence of Fe_2_O_3_ on the flavonoid content and antioxidant capacity of *D. kotschyi*. The application of Fe_2_O_3_ NPs at the level of 50 mg L^−1^ yielded the highest values in terms of flavonoids content and antioxidant activity. A similar observation was made by Asadi-Kavan et al. (2023), who noted an increase in the antioxidant capacity of *O. biennis* following the application of Fe_2_O_3_ NPs. Ghorbanpour (2015) also reported that the use of high levels of iron chelates resulted in an enhancement of flavonoids content in *Carthamus tinctorius* L. Additionally, Kalisz et al. (2021) reported positive effects on the phenol and flavonoid levels, as well as the antioxidant capacity of *C. annuum* seedlings when exposed to Fe_2_O_3_ NPs through foliar application. Azimi et al. (2021) also showed that applying foliar Se NPs to *Dracocephalum moldavica* L. led to increased levels of phenolic compounds and enhanced ROS scavenging. Se affects the pathways involved in phenolic production, notably the shikimic acid pathway, as discussed by Memari-Tabrizi et al. (2021). The higher reactivity of nanomaterials, specifically Fe_2_O_3_ NPs, appears to have a significant impact on enzyme activity and antioxidant capacity. It has been observed that NPs can act as catalysts and induce intracellular chemical alterations (Asadi-Kavan et al., 2020). The application of iron NPs has been shown to increase the flavonoid levels in *D. kotschyi* by promoting the expression of genes involved in the phenylpropanoid biosynthesis pathway (Nourozi et al., 2019). PAL activity in the shikimate pathway triggers the biosynthesis of phenolic metabolites and their precursors in plants. Iron is an essential nutrient for plants as it is necessary for biological redox systems and is an indispensable component of enzymes involved in the biosynthesis of both primary and secondary plant metabolites. Co-factors such as 2-oxoglutarate, Fe^2+^, and ascorbate are required for the catalysis of a series of oxidation reactions in the flavonoid biosynthesis pathway by enzymes like flavones synthase I, flavonol synthase, flavanone 3-hydroxylase, and anthocyanidin synthase/leucoanthocyanidin dioxygenase (Busche et al., 2021). In this study, it was found that the use of Fe_2_O_3_ bulk supplement was more effective than Fe_2_O_3_ NPs in increasing PAL activity. The highest activity of PAL was observed at a level of 200 mg L^−1^ of bulk Fe_2_O_3_. However, it should be noted that changes in PAL activity induced by elicitors do not always correspond to changes in the accumulation of phenolic and flavonoid compounds (Ling et al., 2023). A previous study on *D. kotschyi* demonstrated that the expression of genes involved in PAL and rosmarinic acid synthase increased in response to treatment with Fe NPs (Nourozi et al., 2019). Similarly, in another study on *O. basilicum*, exposure to Fe_2_O_3_ NPs significantly doubled the activity of PAL in the leaves (Ghaffarzadeh et al., 2021).

The excessive generation of ROS such as ˙O_2_^−^, ˙OH, and H_2_O_2_, caused by abiotic factors, leads to oxidative stress. Even under optimal conditions, various metabolic processes in biological systems generate ROS (Mumivand et al., 2021b). H_2_O_2_, produced internally through physiological and pathological processes, is responsible for over 200 genotoxic effects (Villatoro-Pulido et al., 2012). It directly and indirectly damages DNA by generating hydroxyl radicals through a Fenton-type reaction, resulting in oxidative DNA damage, DNA single-strand breaks, gene mutations, and chromosomal aberrations (Luca and Miron, 2016). To counteract the harmful effects of ROS, antioxidant enzymes like APX, CAT, and POX play a significant role in regulating ROS levels (Das and Roychoudhury, 2014; Mumivand et al., 2021a). In this study, both nano and bulk Fe_2_O_3_ supplements induced the production of H_2_O_2_ and subsequently enhanced the activity of enzymatic antioxidants (CAT, APX, and POD) in *D. kotschyi*. This increased antioxidant enzyme activity can help to detoxify H_2_O_2_ and improve the plant's tolerance to oxidative stress (Mittler, 2002). Upon evaluating the functions of all the enzymes examined, it becomes clear that the accumulation of Fe triggers robust antioxidant responses in *D. kotschyi*, thereby facilitating plant growth. In a study by Haydar et al. (2022), higher levels of POD and CAT activity were observed in *Vigna radiata* (L.) R.Wilczek treated with 10 ppm of iron-chelate NP, which supports our findings. Hu et al. (2017) demonstrated excessive ROS production in *Citrus maxima* (Burm.) Merr. induced by Fe_2_O_3_, accompanied by increased production of antioxidant enzymes such as POD and CAT. Insufficient iron as a cofactor in the antioxidant enzyme structures diminishes their effectiveness and makes the plant more vulnerable to environmental stresses (Becana et al., 1998). In a study by Ranieri et al. (2001), it was reported that the activities of POD iso-2enzymes in soybean and sunflower leaves were significantly reduced when grown in a nutrient solution lacking iron. This decrease in activity negatively impacted the plants' ability to scavenge H_2_O_2_. Singh et al. (2021b) demonstrated that the application of 25 ppm Fe_2_O_3_ NPs had a significant positive impact on the morphophysiological and biochemical properties of *E. tereticornis*, alleviating oxidative stress.

Fe_2_O_3_ NPs can enter plant cells through both apoplastic and symplastic routes, where they interact with cellular structures and influence metabolic processes (Tombuloglu et al., 2020). Their unique properties, including hydrogen bonding and ionic interactions, enable these nanoparticles to engage effectively with plant proteins and enzymes, potentially altering biochemical functions and metabolic pathways (Alabdallah and Kotb, 2024; Ali et al., 2018). Recent research has shown that Fe_2_O_3_ NPs can specifically adsorb onto proteins and enzymes, potentially modifying their functions and significantly affecting biochemical pathways within plants (Hasan et al., 2023). Moreover, supramolecular interactions, such as hydrogen bonding, ionic interactions, and π-π interactions, can enhance the stability of these nanoparticles and promote their integration into biological systems (Ahmed et al., 2021).

Based on the comparisons and the physicochemical properties of the Fe_2_O_3_ NPs studied, particularly their size and zeta potential, these NPs are within an optimal range for use as non-fertilizer agents. The particle size observed (20–30 nm) aligns with known ranges that positively influence plant growth and development. In contrast, smaller NPs (e.g., 13.1 nm, Haydar et al., 2022) and larger ones (e.g., 60 nm, Wang et al., 2022) exhibit different biological behaviors. Medium-sized NPs, like those examined here, generally enhance nutrient absorption and improve plant growth due to their superior cellular penetration and interaction. Zeta potential is also crucial for determining the colloidal stability of NPs and their interactions within biological systems. The measured zeta potential of -7.30 millivolts falls within the expected range for similar NPs, indicating stability and effective interaction with plant cell surfaces. Compared to NPs with higher zeta potentials, such as -23.7 millivolts (Singh et al., 2021a), the Fe_2_O_3_ NPs in this study may offer more controlled physicochemical interactions, potentially leading to more favorable biological outcomes. In summary, the size and zeta potential of these NPs suggest they are well-suited to positively impact plant growth and development.

In this study, the highest essential oil content and yield was acquired upon foliar spraying with a concentration of 50 and/or 100 mg L^− 1^ of Fe_2_O_3_ NPs. A higher availability of nutrients could increase the essential oil production in medicinal and aromatic plants by improving the chlorophyll content, photosynthetic rate, plant growth characteristics, and the essential oil glandular trichomes (Ostadi et al., 2020). It might be explained that the impact of iron on the secondary metabolite’s biosynthesis could be similar to stress signals and may lead to an enhanced biosynthesis of these compounds. The recorded results align with the findings of Ahmed et al. (2023) in *Solidago virgaurea* L., Hassanpouraghdam et al. (2022) in *Artemisia dracunculus* L., Danaee and Abdossi (2021) in *O. basilicum*, and Nemati Lafmejani et al. (2018) in *M.* × *piperita*. Amooaghaie and Golmohammadi (2017) observed that the increased availability of micro (Fe, Zn, Cu, and Mn) and macronutrients (N and P) following the application of organic fertilizer resulted in a significant increase of the essential oil productivity in *Thymus vulgaris* L. In a study by Sheikhalipour et al. (2021), chitosan-Se NPs enhanced the EO content in *Momordica charantia* L. Gholinezhad (2017) also noted that optimal morphological traites, essential oil content and yield *of Anethum graveolens* L. were attained through the application of nano-iron fertilizer. Such improvements in essential oil content and yield could possibly be attributed to enhancements in growth, photosynthesis, gene expression of enzyme involved in biosynthesis of secondary metabolites, as well as the distribution and size of secretory glands which serve as specific sites for essential oil biosynthesis after NPs application (Ahmad et al., 2018).

Citral, well-known as 3,7-dimethyl-2,6-octadienal, is an aldehyde monoterpene consisting of a combination of *cis* and *trans* isomers called geranial and neral, respectively. The application of 50 mg L^-1^ of Fe_2_O_3_ NPs led to a significant increase in the concentration of geranial. Conversely, a higher supply of Fe_2_O_3_ NPs (200 mg L^-1^) significantly decreased the amount of neral in the essential oil. Geranial and neral exhibit spasmolytic, antimicrobial, insecticidal, anti-inflammatory, analgesic, and chemopreventive effects (Barnes et al., 2007; Spinozzi et al., 2024). These compounds hold significant economic importance in various industries. Geranial is a crucial component in the flavor and fragrance sectors, as well as in the production of consumer products (Chen and Viljoen, 2010). On the other hand, neral has been identified for its anti-inflammatory properties, making it a potential ingredient in functional food products (Liao et al., 2015). Both geranial and neral play a role in the horticultural trade, particularly in the cultivation of ornamental plants like Gerbera (Ünsal and Yazici, 2021). These findings highlight the diverse and valuable applications of geranial and neral across different sectors of the economy. Furthermore, Zheng et al. (2021) demonstrated that geranial exhibited a higher inhibitory effect against fungi such as *Aspergillus flavus*, *Candida albicans*, and *Trichophyton rubrum* compared with neral.

The application of Fe_2_O_3_ at a concentration of 100 mg L^-1^ led to a significant increase in the concentration of δ3-carene in the essential oil of *D. kotschyi*. Additionally, the spraying of Fe_2_O_3_ caused changes in other major constituents of the essential oil, such as methyl geranate and geranyl acetate. Previous studies have demonstrated that the content and composition of essential oils, as well as their bioactive and protective chemical components, are influenced by genetic, environmental, and agronomic factors (Mumivand et al., 2021d). Similar changes in the essential oil constituents have been observed in *M. × piperita* (Nemati Lafmejani et al., 2018), *S. virgaurea* (Ahmed et al., 2023), and *O. basilicum* (Said-Al Ahl and Mahmoud, 2010) upon the use of Fe_2_O_3_ NPs, consistent with the findings of our current research. The precise mechanism through which the application of NPs modulates plant secondary metabolites is still not completely understood. Recent studies combining phytochemical and genomic approaches have shown that NPs can act as triggers for the production of secondary metabolites by various cellular signaling pathways, including calcium flux, mitogen-activated protein kinases, and ROS metabolism. These alterations in signaling pathways can lead to changes in gene expression levels and the activation of metabolic enzymes, ultimately affecting the biosynthesis of secondary metabolites (Ebadollahi et al., 2019). The DXS gene plays a crucial role in regulating the methylerythritol 4-phosphate (MEP) pathway, which is involved in the synthesis of isoprenoids in plants (Pan et al., 2019; Wright et al., 2014). Apart from its role in isoprenoid biosynthesis, the DXS gene has also been implicated in the regulation of iron homeostasis in plants (Wright et al., 2014). Iron is essential for the biosynthesis of geranyl pyrophosphate, a key precursor in terpenoid production. Studies have shown that pyrophosphate, an iron-chelating agent, can stimulate the enterobactin-dependent iron uptake system, indirectly affecting the biosynthesis of geranyl pyrophosphate (Perrotte-Piquemal et al., 1999). Furthermore, it has been demonstrated that Fe^2+^ can enhance the hydrolysis of geranyl pyrophosphate, potentially influencing its availability for terpenoid production (Vial et al., 1981). The role of pyrophosphate in iron uptake and transport further suggests a direct link between iron and the biosynthesis of geranyl pyrophosphate (De Zwart et al., 2004).

## 5 Conclusions

The current study demonstrated that foliar application of both nano and bulk forms of Fe_2_O_3_ led to increased biomass and enhanced yield attributes in *D. kotschyi*. Both forms of Fe_2_O_3_ acted as growth stimulants, but the Fe_2_O_3_ NPs were particularly effective, showing greater improvements in plant growth, biomass, and tissue biofortification compared to the bulk form. Specifically, Fe_2_O_3_ in its bulk form improved the RWC of leaves. The application of 200 mg L^-1^ of Fe_2_O_3_ NPs and all tested concentrations of bulk Fe_2_O_3_ increased photosynthetic pigments. Our findings highlight the positive effects of Fe_2_O_3_ on flavonoids content and antioxidant activity in *D. kotschyi* whose highest values were observed with 50 mg L^-1^ of Fe_2_O_3_ NPs. The peak activity of PAL occurred at 200 mg L^-1^ of bulk Fe_2_O_3_. Both nano and bulk Fe_2_O_3_ treatments also triggered the production of H_2_O_2_, which in turn enhanced the activity of key enzymatic antioxidants (CAT, APX, and POD) in *D. kotschyi*. Regarding essential oil production, foliar application of Fe_2_O_3_ NPs at 50 and/or 100 mg L^-1^ yielded the highest essential oil content and yield. The 50 mg L^-1^ concentration of Fe_2_O_3_ NPs significantly increased the concentration of geranial in the essential oil. Conversely, a higher Fe_2_O_3_ NP concentration (200 mg L^-1^) significantly reduced the percentage of neral. Applying Fe_2_O_3_ at 100 mg L^-1^ significantly raised the concentration of δ3-carene. Additionally, Fe_2_O_3_ application led to alterations in other major essential oil constituents, including methyl geranate and geranyl acetate. Overall, the foliar spraying of Fe_2_O_3_ NPs not only enhanced the qualitative and quantitative characteristics of *D. kotschyi*, including growth parameters, yield attributes, leaf/steam ratio, essential oil content, and yield, but also minimized the environmental impact. Therefore, we recommend the application of 100 mg L^-1^ of Fe_2_O_3_ NPs as an efficient and ecofriendly treatment for the cultivation of *D. kotschyi*.

## Data Availability

The original contributions presented in the study are included in the article/**Supplementary Material**. Further inquiries can be directed to the corresponding author.
