# Supplementary material for: Application of Fe2O3 nanoparticles improves the growth, antioxidant power, flavonoid content, and essential oil yield and composition of Dracocephalum kotschyi Boiss
Source: Front Plant Sci. 2024 Oct 9;15:1475284. doi: 10.3389/fpls.2024.1475284 (PMC11500079; doi:10.3389/fpls.2024.1475284)
Supplement: Supplementary file 1 [file Table1.docx]

| Supplementary Table 1. Fe_2_O_3_ NPs specifications used in the experiment and Colloidal Properties of as-prepared nano-metal oxides particles | | | | | |
| --- | --- | --- | --- | --- | --- |
| Property | nano-Fe_2_O_3_ | Compounds | percent |  |  |
| Shape | spherical | Ca | < 0.0229 | Zeta potential (mV ) | -7.30 |
| Purity (percent) | 98+% | Cr | < 0.0016 | Polydispersity Index (PdI) | 0.454 |
| Outer diameter (nm) | 20 - 30 | K | < 0.0012 | Hydrodynamic Diameter (nm) | 203.9 |
| Bulk Density (g / cm^3^) | 0.84 | Mn | < 0.0860 |  |  |
| True Density | 4.8-5.1 | SiO_2_ | < 0.1420 |  |  |
| Color | Dark Brown |  |  |  |  |

| Supplementary Table 2. Some physicochemical properties of pot substrate | | | | | |
| --- | --- | --- | --- | --- | --- |
| No | Soil properties | Unit | No | Soil properties | Unit |
| 1 | Total nitrogen (%) | 0.23 | 9 | Organic carbon (%) | 1.17 |
| 2 | Available potassium (mgkg^-1^) | 277 | 10 | pH | 7.7 |
| 3 | Available phosphorus (mg.kg^-1^) | 6.6 | 11 | EC (ds/m) | 2.03 |
| 4 | Iron (mg.kg^-1^) | 1.71 | 12 | Sand (%) | 55.6 |
| 5 | Magnesium (mg.kg^-1^) | 99.4 | 13 | Clay (%) | 19.2 |
| 6 | Zinc (mg.kg^-1^) | 0.83 | 14 | Silt (%) | 25.2 |
| 7 | Copper (mg.kg^-1^) | 0.41 | 15 | Calcium carbonate (%) | 7.8 |
| 8 | Boron (mg.kg^-1^) | 0.41 |  |  |  |

| Supplementary Table 3. Variance analysis of the effects of Fe_2_O_3_NPs and bulk Fe_2_O_3_ foliar application on biomass and yield attributes of *D. kotschyi* | | | | | | | | | | | | |
| --- | --- | --- | --- | --- | --- | --- | --- | --- | --- | --- | --- | --- |
| Sov | Df | Plant height | Inflorescence length | Leaf length | Leaf width | Stem diameter | Internode length | Plant fresh  weight | Plant dry  weight | Leaf dry  weight | Stem dry  weight | Leaf/Stem ratio |
| Fe_2_O_3_ | 6 | 0.0087^**^ | 32.89^**^ | 8.51^**^ | 0.84^ns^ | 0.135^**^ | 70.94^**^ | 2.32^ns^ | 0.38^ns^ | 1.73^**^ | 0.89^**^ | 0.97^**^ |
| Error | 14 | 0.0007 | 0.98 | 1.63 | 1.75 | 0.027 | 0.84 | 1.107 | 0.503 | 0.031 | 0.089 | 0.047 |
| Cv |  | 3.54 | 8.71 | 5.64 | 7.37 | 8.82 | 3.03 | 7.62 | 14.85 | 6.73 | 17.46 | 13.08 |
| **: Significant at 1% level, *: Significant at 5% level, ns: not statistically significant. | | | | | | | | | | | | |

| Supplementary Table 4. Variance analysis of the effects of Fe_2_O_3_NPs and bulk Fe_2_O_3_ foliar application on biochemical traits and Fe concentration of *D. kotschyi* | | | | | | | | | | |
| --- | --- | --- | --- | --- | --- | --- | --- | --- | --- | --- |
| Sov | Df | Relative water content | Chlorophyll a | Chlorophyll b | Total chlorophyll | Carotenoid | Total phenol | Total flavonoids | FRAP assay | Fe concentration |
| Fe_2_O_3_ | 6 | 77.43^**^ | 0.044^**^ | 0.0103^**^ | 0.124^**^ | 6.51^**^ | 0.00019^**^ | 195.08^**^ | 1.062^**^ | 57736.96^**^ |
| Error | 14 | 2.24 | 0.0020 | 0.00086 | 0.008 | 0.42 | 0.00001 | 3.56 | 0.075 | 419.94 |
| Cv |  | 3.46 | 4.82 | 10.72 | 7.42 | 7.26 | 1.16 | 4.20 | 8.75 | 10.65 |
| **: Significant at 1% level, *: Significant at 5% level. | | | | | | | | | | |

| Supplementary Table 5. Variance analysis of the effects of Fe_2_O_3_NPs and bulk Fe_2_O_3_ foliar application on antioxidant enzymes and PAL activity, H_2_O_2_ and essential oil content and yield of *D. kotschyi* | | | | | | | | |
| --- | --- | --- | --- | --- | --- | --- | --- | --- |
| Sov | Df | Ascorbate peroxidase (APX) | Peroxidase (POD) | Catalase (CAT) | [Phenylalanine ammonia lyase](https://scholar.google.com/scholar?q=Phenylalanine+ammonia+lyase&hl=fa&as_sdt=0&as_vis=1&oi=scholart) (PAL) | Hydrogen peroxide (H_2_O_2_) | Essential oil  content (%) | Essential Oil Yield |
| Fe_2_O_3_ | 6 | 7337.81^**^ | 155.32^**^ | 49.26^**^ | 257.34^**^ | 11.22^**^ | 0.308^**^ | 0.00053^**^ |
| Error | 14 | 69.27 | 6.763 | 0.29 | 5.949 | 0.53 | 0.031 | 0.000036 |
| Cv |  | 15.38 | 14.24 | 14.76 | 20.017 | 13.68 | 10.57 | 8.47 |
| **: Significant at 1% level, *: Significant at 5% level. | | | | | | | | |

| Supplementary Table 6. Variance analysis of the effects of Fe_2_O_3_NPs and bulk Fe_2_O_3_ foliar application on *D. kotschyi* essential oil constituents | | | | | | | | | | | | |
| --- | --- | --- | --- | --- | --- | --- | --- | --- | --- | --- | --- | --- |
| Sov | Df | *α*-Pinene | Camphene | Sabinene | *β*-Myrcene | *α*-Phellandrene | *3*-Carene | *cis*-Sabinene hydrate | Terpinolene | Linalool | *α*-Campholenal | *E*-2,6-Nonadien-1-ol |
| Fe_2_O_3_ | 6 | 0.55^ns^ | 0.00025 ^ns^ | 0.015^ns^ | 0.17^ns^ | 0.017^ns^ | 11.13^**^ | 0.031^**^ | 0.0049^ns^ | 0.041^ns^ | 0.026^**^ | 0.010^**^ |
| Error | 14 | 0.677 | 0.00016 | 0.016 | 0.084 | 0.0073 | 0.311 | 0.0074 | 0.0031 | 0.020 | 0.0068 | 0.0014 |
| Cv |  | 16.31 | 15.87 | 14.77 | 12.89 | 20.75 | 7.14 | 20.6 | 14.95 | 13.07 | 14.95 | 9.35 |
| **: Significant at 1% level, *: Significant at 5% level, ns: not statistically significant. | | | | | | | | | | | | |

| Continue Supplementary Table 6. Variance analysis of the effects of Fe_2_O_3_NPs and bulk Fe_2_O_3_ foliar application on the essential oil constituents of *D. kotschyi* | | | | | | | | | | | | | |
| --- | --- | --- | --- | --- | --- | --- | --- | --- | --- | --- | --- | --- | --- |
| Sov | Df | *cis*-Verbenol | *iso*-Pulegone | 1,3,4-Trimethyl-3-cyclohexenyl-1-carboxaldehyde | *γ*-Terpineol | Neral | Geranial | Methyl geranate | Geranyl acetate | (*E*)-Caryophyllene | Aromandendrene | *β*-Gurjunene |  |
| Fe | 6 | 0.012^*^ | 0.155^**^ | 0.42^**^ | 0.014^ns^ | 5.033^**^ | 6.46^*^ | 4.44^**^ | 10.067^*^ | 0.0006^**^ | 0.004^**^ | 0.133^**^ |  |
| Error | 14 | 0.0036 | 0.014 | 0.036 | 0.0074 | 0.63 | 2.24 | 0.86 | 3.24 | 0.000009 | 0.0005 | 0.014 |  |
| Cv |  | 22.68 | 12.62 | 11.68 | 13.81 | 3.62 | 4.24 | 13.59 | 16.58 | 39.29 | 34.33 | 16.49 |  |
| **: Significant at 1% level, *: Significant at 5% level, ns: not statistically significant | | | | | | | | | | | | | |
